# 1730. Real-World Use Of Dalbavancin In A US Tertiary Referral Center

**DOI:** 10.1093/ofid/ofac492.1360

**Published:** 2022-12-15

**Authors:** Mudita Bhugra, Aaron Chang, James Polega, Jorgelina De Sanctis, Derek Vanderhorst, Habiba Hassouna

**Affiliations:** Spectrum Health /Michigan State University, Grand Rapids, Michigan; Michigan State College of Human Medicine, Grand Rapids, Michigan; Spectrum Health/Michigan State University, Grand Rapids, Michigan; Spectrum Health/Michigan State University, Grand Rapids, Michigan; Spectrum Health, Grand Rapids, Michigan; Spectrum Health/Michigan State University, Grand Rapids, Michigan

## Abstract

**Background:**

Dalbavancin is a lipoglycopeptide with prolonged half-life currently approved for treatment of bacterial skin and soft tissue infections. Off-label uses of dalbavancin include multiple Gram-positive infections requiring long-term antibiotics use. However, clinical data regarding Dalbavancin use in the real-world setting remains limited.

**Methods:**

We conducted a retrospective cohort study of all adult inpatients who were administered ≥1 dose of Dalbavancin between November 2017 and March 2022.

**Results:**

Forty-nine adults were identified. Dalbavancin was used to treat skin/soft tissue infections in 9 patients (18.8%). Off-label uses accommodated for the majority of patients, with diagnoses included: bloodstream infection (24, 50%), osteomyelitis (9,18.8%), native valve infective endocarditis (6,12.5%), native septic arthritis (6,12.5%), epidural abscess (4,8.3%), catheter-related bacteremia (3,6.3%) prosthetic joint infection (3,6.3%), and diabetic foot infection (1,2.1%). No patients with prosthetic valve infective endocarditis were identified. Staphylococcus aureus was the most common treated pathogen: MRSA (15, 35.7%), MSSA (17, 40.5%). Other pathogens included: Streptococcus (2,4.8%), Enterococcus (4,9.5%), coagulase negative Staphylococcus (5,11.9%), other gram positive (4,9.5%), none (2,4.8%). Among patients who completed therapy, overall cure, and clinical response as assessed at day 42 was achieved in 29 (96.7%) of patients,1 patient (3.3%) had relapse due to noncompliance with antimicrobial suppressive regimen. Intravenous drug use was the most common cause among patients who did not complete treatment (10, 58.8%). Adverse events included mild elevation in liver function tests, which were reversible and were not definitively related to the treatment. No rashes or infusion related reaction were reported. There were no adverse events resulting in drug discontinuation.

**Conclusion:**

Real-world, including off-label, use of Dalbavancin appears safe and is associated with favorable treatment responses. Therefore, it should be considered as an alternative treatment approach in certain patient population including at risk population that may otherwise discharge from the hospital with no antimicrobial treatment or suboptimal oral antibiotics.

**Disclosures:**

**All Authors**: No reported disclosures.

